# An interpretable multiple-instance approach for the detection of referable diabetic retinopathy in fundus images

**DOI:** 10.1038/s41598-021-93632-8

**Published:** 2021-07-12

**Authors:** Alexandros Papadopoulos, Fotis Topouzis, Anastasios Delopoulos

**Affiliations:** 1 Multimedia Understanding Group, Information Processing Laboratory, Department of Electrical and Computer Engineering, Aristotle University of Thessaloniki, Thessaloniki, Greece; 2 Department of Ophthalmology, Aristotle University Medical School, Thessaloniki, Greece

**Keywords:** Retinal diseases, Computer science

## Abstract

Diabetic retinopathy (DR) is one of the leading causes of vision loss across the world. Yet despite its wide prevalence, the majority of affected people lack access to the specialized ophthalmologists and equipment required for monitoring their condition. This can lead to delays in the start of treatment, thereby lowering their chances for a successful outcome. Machine learning systems that automatically detect the disease in eye fundus images have been proposed as a means of facilitating access to retinopathy severity estimates for patients in remote regions or even for complementing the human expert’s diagnosis. Here we propose a machine learning system for the detection of referable diabetic retinopathy in fundus images, which is based on the paradigm of multiple-instance learning. Our method extracts local information independently from multiple rectangular image patches and combines it efficiently through an attention mechanism that focuses on the abnormal regions of the eye (i.e. those that contain DR-induced lesions), thus resulting in a final image representation that is suitable for classification. Furthermore, by leveraging the attention mechanism our algorithm can seamlessly produce informative heatmaps that highlight the regions where the lesions are located. We evaluate our approach on the publicly available Kaggle, Messidor-2 and IDRiD retinal image datasets, in which it exhibits near state-of-the-art classification performance (AUC of 0.961 in Kaggle and 0.976 in Messidor-2), while also producing valid lesion heatmaps (AUPRC of 0.869 in the 81 images of IDRiD that contain pixel-level lesion annotations). Our results suggest that the proposed approach provides an efficient and interpretable solution against the problem of automated diabetic retinopathy grading.

## Introduction

Diabetic retinopathy (DR) is a complication of diabetes mellitus that can lead to blindness if left untreated. More than 1 out of 3 diabetic patients are expected to develop DR during their lifetime^1^, with their chances increasing over time^2^. Yet, despite its prevalence among diabetic populations, the risk of blindness can be significantly reduced via timely treatment, i.e. before the retina is severely damaged^3^.

Diabetic retinopathy is characterized as either non-proliferative (NPDR), meaning it manifests mainly through retinal lesions, or as proliferative (PDR), meaning neovascularization of weak blood vessels also occurs. The International Clinical Diabetic Retinopathy Disease (ICDR) severity scale^4^, suggests a finer classification of the disease into the following 5 stages, based on the observable findings during eye examination: 1. No apparent retinopathy 2. Mild NPDR 3. Moderate NPDR 4. Severe NPDR 5. PDR. Common guidelines recommend annual screenings for diabetic patients without or with mild DR, 6 month follow up examination for moderate DR, and referral to an ophthalmologist for treatment evaluation for severe cases^5^. When DR is beyond the mild stage ($\ge$ moderate) or when Diabetic Macular Edema (DME) is present, the disease is further characterized as *referable diabetic retinopathy (rDR)*. Subjects who were diagnosed with rDR by a trained DR grader (not necessarily an ophthalmologist) must also be referred to an ophthalmologist for further evaluation of their condition.

Screening for DR is usually carried out by either an in-person eye examination or by means of retinal photography. In either case, an eye care professional examines the retina (either directly through a slit-lamp or indirectly through a high resolution retinal photograph captured with a specialized camera) for signs of the disease, such as microaneurysms, haemorrhages and hard or soft exudates. Other factors such as macular edema, narrowing of the blood vessels or damage in the nerve tissue are also considered^3^. In general, accurate DR grading is a daunting task even for experienced graders, and, as a result, inter-grader variability is quite common^6^.

In recent years, retinal photography has been widely accepted as an adequate screening method that can even lead to improved diagnostic performance compared to the standard slit-lamp examination^7^. This acceptance has naturally led to the conduction of much research on the development of automated methods for grading retinal images, as such techniques can provide substantial benefits to the standard DR screening procedure. For example, they can support retinal specialists by lightening their workload or by identifying cases they might have missed. Automated grading can also be used to increase the coverage of nation-wide screening programs by facilitating access for people in remote or rural regions via the use of teleophthalmology.

Image classification is a classic use case for machine learning (ML) algorithms. especially those based on the paradigm of deep learning. In recent years, deep learning has achieved impressive results in a series of tasks, including image classification. In this work, we propose a method based on deep learning to detect referable diabetic retinopathy from retinal images and simultaneously produce heatmaps of the most dominant DR lesions to aid the model’s interpretability. Our method operates by independently extracting information from multiple small image patches and combining them based on their content. We focus on the detection of rDR, as this simplified binary classification task is a common target for automated DR grading methods that provides ample clinical utility^8^.

Our paper is organized in the following way: first, we review the most prominent works in the related literature. Then, we introduce the proposed methodology for performing rDR detection and lesion localization. We provide details about the datasets used in our experiments and then, we present the experimental results for our method, as well as comparisons with other works in the literature. Finally, we discuss our method and results and conclude the paper.

## Related work

The automated analysis of retinal images for diabetic retinopathy detection has received impressive research attention over the last decade, particularly following the rise of deep learning that eliminated the need of manually constructing problem-specific features. Many different learning tasks have been pursued in the related literature, such as optic disk and blood vessel segmentation, lesion detection and DR grading. As the relevant literature is abundant, in this section we will focus on the tasks of DR grading and lesion detection that are mostly related to our work. For a thorough review of the field, we refer the reader to the excellent survey of Stolte et al.^9^.

In grading approaches, the task is usually cast as a binary classification problem of no rDR vs rDR, but it can be also viewed as a multi-class problem, using the stages of the ICDR severity scale as 5 distinct classes, Early methods relied on traditional computer vision methods, mainly using feature extraction techniques to identify specific properties of interest in the fundus image, coupled with shallow machine learning models for classification. For instance, Nayak et al.^10^ used morphological and texture analysis to extract blood vessel and hard exudate features which were then used as input features in a neural network, Acharya et al.^11^ made use of higher-order spectra coupled with an SVM classifier and, more recently, Seoud et al.^12^ proposed a set of high-performing, handcrafted shape features that were fed to a Random Forest classifier.

In recent years, the rise of *Deep Convolutional Neural Networks (DCNN)* and their dominance in computer vision tasks has transformed the research landscape, so that most works are nowadays based on deep neural networks. The trend reached its peak with the seminal work of Gulshan et al.^13^ that reported excellent rDR classification performance (ROC AUC of 0.99), by using an Inception v3 model pre-trained on ImageNet and finetuned on a private dataset of 120k fundus images. A replication study using the publicly-available Kaggle-EyePACS dataset^14^, was conducted by Voets et al.^15^, but was unable to reproduce the results of the original study, suggesting that the ground-truth quality is a decisive factor for achieving high performance, as the dataset used in the original study was annotated by a panel of 7 retina specialists, while Kaggle-EyePACS was annotated by a single specialist. This is further corroborated by the findings of Krause et al.^6^, who report performance improvements when using even a small amount of adjudicated DR ground truths.

The work of Pires et al.^16^ investigates the use of a multi-resolution training scheme, using different networks with shared weights for each different resolution of the input image. They also extract and combine features simultaneously from multiple label-preserving random perturbations of the same input image, as suggested by the team that placed second (https://tinyurl.com/s9jz442y) in the Kaggle DR competition, along with other tricks, such as aggressive data augmentation, to improve performance.

An interesting addition to the standard DR prediction procedure is suggested by Leibig et al.^17^. In particular, they incorporate a bayesian estimation of the model’s uncertainty during test time, implemented via a dropout approximation^18^. They go on to show, that by refraining from classifying images for which the model is uncertain, one can increase classification performance and obtain more reliable predictions. This modification is applicable to any of the usual deep CNN models that have been suggested in the context of DR classification, such as ResNet^19^, AlexNet^8^ and VGG^16^.

A different strategy that is more on par with the screening procedure of eye specialists is to first detect specific DR lesions and then use these detections to infer DR. Following this direction, Abramof et al.^8^ introduced a series of lesion detector models, based on AlexNet^20^ and VGG^21^, where each detector is applied to the input image to detect a particular type of DR lesion (haemorrhages, exudates, etc). The detector outputs are then fused together to form a feature vector, that is used to first assess the image quality, and then, if the quality is found acceptable, to predict the level of DR. The resulting hybrid system was shown to achieve very high performance and has been deployed in real world conditions.

Such methods, however, depend on the manual annotation of DR lesions, a procedure that significantly increases the clinician workload and, as such, can be typically be carried out for few images. In addition, disease-relevant information tend to be located in just a few regions in the input image^22^, so that using a single feature vector to represent the entire image can be problematic. To aid in these problems, *Multiple-Instance Learning (MIL)* methods^23^ that treat the input image as bag of patches accompanied by a single DR label, have been suggested. An early MIL method introduced by Quellec et al.^24^, used rectangular patches originating from DR-positive images to train a model using a patch relevance criterion. The resulting model was then shown to produce high relevance scores for patches that contained DR lesions. The subsequent work of Kandemir et al.^22^ employed a traditional MIL pipeline, in which each patch was represented with a set of handcrafted features (intensity histograms and texture features). The resulting bags of patch features were then classified using a variety of shallow MIL algorithms, like the mi-SVM method^25^, and the best performing one was deemed the most suitable. A similar approach that made use of deep learning instead of handcrafted features was later proposed by Zhou et al.^26^, who proposed a modified version of AlexNet to produce per-patch DR predictions, which were then aggregated according to some pre-defined aggregated criterion and thresholded to reach a final DR prediction for the input image.

Here we introduce a method to perform rDR classification by analyzing the fundus image on the patch level. Our approach follows the MIL paradigm that treats each input image as a bag of image patches. At first, each patch is independently encoded via a deep CNN model into a feature vector. Contrary to other MIL approaches in which the individual patch vectors are pooled via pre-defined operators, here we examine the use of the attention mechanism^27^. In the context of MIL, attention serves as a trainable pooling operator that learns to put emphasis on the most informative instances (i.e. patches that contain DR lesions) and ignore the uninformative ones, thus leading to an image-level representation that retains the most relevant information and can thus be used for efficient rDR detection.

In medical applications, model interpretability is key for the successful adoption of a proposed ML solution, as human experts are more likely to trust a decision if they understand the motives behind it. In the case of DR in particular, a model is usually considered black-box during training and endowed with intrepretability properties using some post-training optimization procedure to output a heatmap of the fundus regions^19, 28, 29^ that the model is most sensitive to. In this work, we follow a different approach: we leverage the attention mechanism to construct heatmaps that highlight the image regions that directly contributed to a given prediction. In doing so, heatmap generation arises quite naturally since attention is a basic building block of our model.

We evaluate the classification performance of our model on the publicly available Kaggle-EyePACS^30^ and Messidor-2^8^ datasets. In the rDR detection task, an ensemble of 5 models trained on the standard Kaggle-EyePACS training set, achieves a ROC AUC of 0.961 on the Kaggle-EyePACS test set and 0.976 on Messidor-2, outperforming other patch-based methods and performing on-par with the state-of-the-art. We also perform experiments to evaluate the quality of the produced attention heatmaps using the images from the IDRiD^31^ dataset that contain detailed pixel-level lesion annotations. Our results in these experiments show that the attention mechanism can efficiently recognize the DR-induced lesions in the fundus of the eye.

### Methods

### Image pre-processing

A practical DR grading system must be able to handle fundus images of different resolutions, lighting conditions and view points of the retina disk, as these parameters may vary between different retinal cameras and capturing conditions. However, typical image classification models operate on images of pre-defined dimensions. In addition, artificial artifacts in the image, such as blobs caused by dust in the lens or by improper lightning, can seriously hinder model performance. Therefore, to mitigate these issues we apply a common pre-processing step to all images prior to any machine learning analysis. The pre-processing pipeline consists of the following steps:

1. Estimate the center and radius of the circular eye disk by means of the Hough transform (can be skipped if ROIs are provided by the retinal camera).
2. Crop a rectangular region with edge equal to the estimated eye disk radius and centered at the estimated disk center.
3. Resize the rectangular crop to a common resolution of $512\times 512$.
4. Subtract the local color average as suggested in Graham et al.^14^, to account for the variability in lightning conditions across images.
5. Zero-out the outer 5% of the retina disk to remove artificial boundary effects introduced by the filtering procedure.

**Figure 1:**
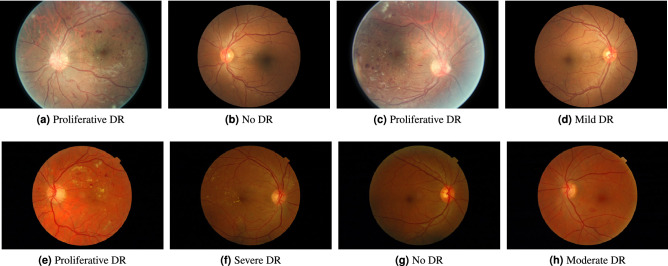
Sample images from the Kaggle-EyePACS dataset (top row) and the Messidor-2 dataset (bottom row) with their corresponding DR grades. The first row also showcases the two types of fundus images contained in Kaggle-EyePACS, i.e. images where the circular disk of the eye is fully captured (right column) or images where the circular disk is partially clipped (left column).

**Figure 2:**
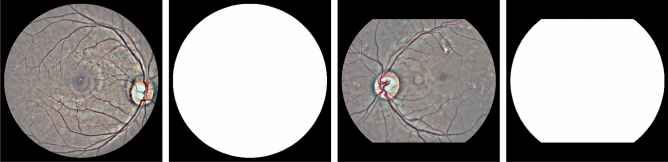
Samples of pre-processed retinal images (left) along with their corresponding binary retina masks (right).

Images for which the disk center cannot be found via the Hough transform are discarded altogether. Additional care is taken for images that do not contain the whole circular disk (for example Fig. 1a,c). In such cases, an additional zero padding step of appropriate size is applied along the height dimension, before cropping the rectangular retina region. The overall process results into two components that will used in the subsequent learning pipeline: the preprocessed image, which is ready to be fed to a machine learning model, and the binary retina mask (obtained during the Hough transform step) which can be used to separate the retina from the image background. Samples of preprocessed images and their corresponding retina masks are given in Fig. 2.

### Bag of patches encoding

Having established a common frame of reference for all images, we now compute the bag of patches representation for an image. To this end, rectangular patches of size $d\times d$ pixels are extracted from each image. We use different policies for patch extraction during training and testing. During training we randomly select $K_{o}$ patches from the pool of possible image patches. This significantly speeds up training as we can represent an image using only a small subset of all the possible patches and also adds a regularization effect, as the representation of a specific image will be different each time it is fed to the network (since it will be represented by a set of different random $K_{o}$ patches). At test time, we perform exhaustive patch extraction over a regular grid with a patch overlap ratio of $t\in [0,1)$. In both cases, some patches will contain very small part of the retina or originate completely from the image background. To avoid such cases, we use the previously computed retina mask and discard patches whose eye disk content is less than $50\%$. The surviving *K* patches form a set $X=\{{\mathbf {x}}_1, {\mathbf {x}}_2, \dots , {\mathbf {x}}_K\}$ with ${\mathbf {x}}_k \in {\mathbb {R}}^{d \times d \times 3}$ that constitutes the bag of patches for the given image. In the following, we will discuss how we can efficiently utilize the bag of patches representation to perform rDR prediction.

**Figure 3:**
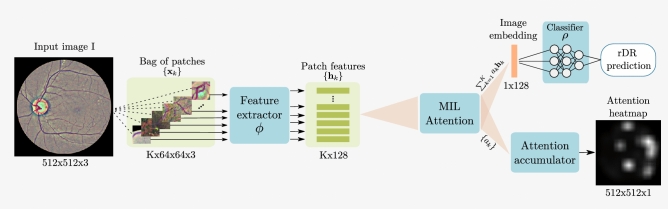
High-level overview of the proposed rDR prediction pipeline. The preprocessed image *I* is represented by K rectangular patches with fixed overlap that cover most of the retinal disk (patches that have less than 50% retinal content, such as the patches extracted near the boundary of the retinal disk, are discarded). Features are then extracted from each ${\mathbf {x}}_k$ patch via a Resnet-18 (function $\phi$) model Each resulting feature vector ${\mathbf {h}}_k$ is assigned a weight $\alpha _k$ by an attention mechanism and the weighted average of all the vectors $\{{\mathbf {h}}_k\}$ is used to acquire a single embedding that describes the entire image. Finally, the embedding is fed to a fully connected layer (function $\rho$) that outputs the probability of rDR for the given image. At the same time, the attention weights of different patches are mapped to their corresponding image regions and an attention heatmap that highlights the most salient image regions according to the model is constructed.

### Multiple-instance modelling

In the MIL view of the rDR detection problem, we are essentially interested in learning a function *f* that will map any set $X=\{{\mathbf {x}}_1, {\mathbf {x}}_2, \dots , {\mathbf {x}}_K\}$ with ${\mathbf {x}}_k \in {\mathbb {R}}^N$ (corresponding to the bag of patches of the input image) to a real number (corresponding to the probability of rDR for that image). Being a set function, *f* must be invariant to the different permutations of *X*. Previous theoretical work^32^ suggested that any permutation-invariant function over a set *X* can be modelled as a sum decomposition of the form $\rho \left( \mathop {\sum }_{{\mathbf {x}} \in X}\phi \left( {\mathbf {x}} \right) \right)$, where the transformation $\phi :{\mathbb {R}}^{N} \mapsto {\mathbb {R}}^M$ is applied elementwise to each set instance, thus leading to a transformed set $H= \{{\mathbf {h}}_1, {\mathbf {h}}_2, \dots , {\mathbf {h}}_K\} = \{\phi ({\mathbf {x}}_1), \phi ({\mathbf {x}}_2), \dots , \phi ({\mathbf {x}}_K),\}$, while the transformation $\rho :{\mathbb {R}}^M \mapsto {\mathbb {R}}$ is applied to the result of summing the elements of *H* to obtain the desired output. Stepping on this result, Ilse et al.^27^ proposed to incorporate the attention-mechanism^33^ in the sum-decomposition, as an elegant way of tackling multiple-instance classification problems. More specifically, they proposed to model the label probability of a bag via the following mechanism:

1 $$\begin{aligned} p(y|X) = \rho \left( {\mathbf {z}} \right) = \rho \left( \sum _{k=1}^{K} \alpha _k {\mathbf {h}}_k \right) = \rho \left( \sum _{k=1}^{K} \alpha _k \phi \left( {\mathbf {x}}_k\right) \right) \end{aligned}$$

where $\phi :{\mathbb {R}}^{N} \mapsto {\mathbb {R}}^M$ and $\rho :{\mathbb {R}}^M \mapsto [0, 1]$. The coefficients $\alpha _k$ in Eq. (1) can be computed via the additive attention^33^ mechanism of Eq. (2), which introduces the learnable parameters ${\mathbf {V}} \in {\mathbb {R}}^{L \times M}, {\mathbf {w}} \in {\mathbb {R}}^{L \times 1}$.

2 $$\begin{aligned} \alpha _k = \frac{\exp {({\mathbf {w}}^T \tanh {({\mathbf {Vh}}_k^T}) } )}{\sum _{k=1}^{K}\exp {({\mathbf {w}}^T \tanh {({\mathbf {Vh}}_k^T}) })} \end{aligned}$$

In this work, we adopt the attention-based multiple-instance scheme for the purposes of detecting rDR in a fundus image. In a nutshell, we propose the following processing pipeline:

1. Pre-process the input image *I* according to the previously described pipeline.
2. Compute its bag of patches representation.
3. Transform the bag of patches $X=\{{\mathbf {x}}_1, {\mathbf {x}}_2, \dots , {\mathbf {x}}_K\}$ with ${\mathbf {x}}_i \in {\mathbb {R}}^{d \times d \times 3}$ to a bag of features $H=\{{\mathbf {h}}_1, {\mathbf {h}}_2, \dots , {\mathbf {h}}_K\}$ with ${\mathbf {h}}_i \in {\mathbb {R}}^M$, by applying the function $\phi$ to each patch.
4. Apply attention pooling to arrive at a global image representation vector ${\mathbf {z}} = \sum _{k=1}^{K} \alpha _k {\mathbf {h}}_k$
5. Estimate the rDR probability for *I* by computing $\rho ({\mathbf {z}})$ (where $\rho$ is modelled by a fully-connected network).

One attractive property of the MIL attention approach is that all transformations can be modelled using deep neural networks and the resulting model can still be trained in an end-to-end manner. This would not be possible if some other commonly used pooling operator, such as Bag of Features or Fisher Vectors, was used. For our specific problem, we elect to use a high capacity CNN (ResNet-18)^34^ to model the feature extraction function $\phi$ and a single fully-connected layer to model the final classifier $\rho$. We chose the ResNet-18 over the more high capacity alternatives, like ResNet-34 or ResNet-50, as they offered no improved performance on the validation set. The attention mechanism is implemented via a fully-connected network with 2 layers that correspond to the parameters ${\mathbf {V}}$ and ${\mathbf {w}}$ of Eq. (2). A schematic overview of the proposed system is given in Fig. 3.

### Interpretable attention heatmaps

After training, we can use the attention mechanism to visualize the fundus regions that affect the model’s decision. As we previously saw, in order to classify an image, we assign a weight to each patch using the attention mechanism. These weights offer an implicit way of identifying which image regions contribute the most to the model’s prediction. Ideally, in true positive cases, the model should assign high weights $\alpha _k$ to patches that contain DR lesions, such as haemorrhages and exudates, while in true negative cases, all patches should receive similar weights. Such a heatmap could also assist in understanding the reasons behind misclassifications, either false positives or false negatives and enable targetted interventions in the learning pipeline.

**Figure 4:**
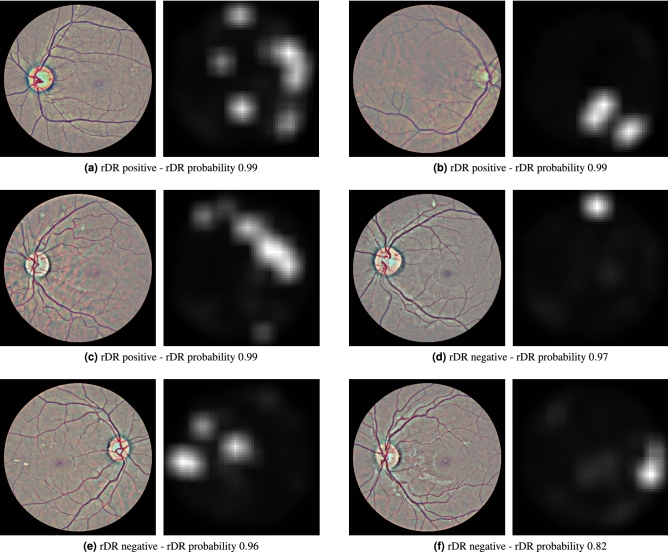
Examples of attention heatmaps produced by the model for 6 Messidor-2 images that corresponded to both true-positive and false-positive predictions.

To produce the attention heatmap, we start with an initially zeroed single-channel image of same height and width as the pre-processed images. This auxiliary image will be used to accumulate the attention values: we iterate the patches and add their assigned weights to the pixels of the accumulator image that correspond to the location of each patch in the original image. The granularity of the pixel-level assignment can be controlled via the patch overlap parameter *t*: larger overlap between the extracted patches leads to more granular and aesthetically pleasing visualizations, while smaller overlap speeds up the computations but results in coarser visualizations. The values of the auxiliary image are then linearly mapped to the [0, 1] range to produce a proper heatmap. Examples of such heatmaps are given in Fig. 4.

## Datasets

### Kaggle-EyePACS

The Kaggle Diabetic Retinopathy Detection^30^ challenge dataset consists of high-resolution retina images captured under a variety of imaging conditions. It contains 88.702 RGB images of differing resolutions (from $433\times 289$ up to $5184\times 3456$) that are partitioned in a training set of 35.126 and a test set of 53.576 images. A clinician has graded all images according to the ICDR scale. The DR grade distribution can be seen in Table 1. Some indicative images from this dataset can be seen in Fig. 1.

It is estimated^35^ that $25\%$ of the Kaggle-EyePACS images are ungradable because they contain artifacts (loss of focus, under or overexposure etc.) that prevent a medical expert from assessing their DR grade. In fact, it is quite common for DR image graders to first evaluate the image quality and proceed with the actual DR grading only if they find it acceptable. The authors of^15^ suggested that image quality estimation does not require medical expertise and can, therefore, be carried out by non-experts They went on to manually evaluate the quality of all images in the Kaggle dataset, based on instructions provided to the professional DR graders for performing the same task in^13^. In doing so, they rejected about $19.9\%$ of the images, resulting in a filtered dataset of 71.056 images. In our experiments, we examine what happens both when adopting these gradability estimates and when using the full training and test sets.

### Messidor-2

The Messidor-2 dataset^8^ contains 1748 fundus images captured with a Topcon TRC NW6 camera in three different resolutions: $1440\times 960$, $2240\times 1488$ and $2304\times 1536$. As there is no official DR ground-truth to accompany the dataset, grades for DR and image quality are available from a variety of sources, such as the University of Iowa (https://tinyurl.com/58sb5rm3), a panel of 7 graders by Google^13^ and a panel of 3 graders again by Google^6^. Image grading by multiple experts means that images that cause disagreements among them are re-examined and adjudication sessions are carried out until consensus is reached. As a result, in terms of label quality, Messidor-2 can be considered less noisy than the Kaggle-EyePACS dataset, in which DR grades are provided by a single DR grader. In this work we use the Google-3 grades for evaluating Messidor-2 per image as they are available for all the 1748 images, and the UIowa grades for evaluating Messidor-2 per patient, as the publicly available version of these grades is limited on the per-patient rDR level. The DR grade distribution for this dataset is given in Table 1, while sample images are provided in Fig. 1.

**Table 1:** Distribution of the 5 DR grades in the images of the Kaggle-EyePACS and Messidor-2 datasets.

Dataset	No rDR		rDR			Split	
	No DR	Mild	Moderate	Severe	Proliferative	Train	Test
Kaggle (all)	65.343 (73.6%)	6.205 (6.9%)	13.153 (14.8%)	2.087 (2.3%)	1.914 (2.1%)	35.126	53.576
Kaggle (gradable)	52.649 (74.1%)	5.073 (7.1%)	10.279 (14.4%)	1.596 (2.2%)	1.372 (1.9%)	28.098	42.871
Messidor-2 (G-3)	1.017 (58.3%)	270 (15.4%)	347 (19.8%)	75 (4.3%)	35 (2.0%)	1.744	
Messidor-2 (UIowa)	684		190			874	

Available grades for Kaggle-EyePACS have been provided by a single expert. For Messidor-2 the publicly available grades include an adjudication by a panel of 3 experts (G-3) and a per-patient diagnosis of rDR versus no rDR (UIowa).

### IDRiD

**Figure 5:**
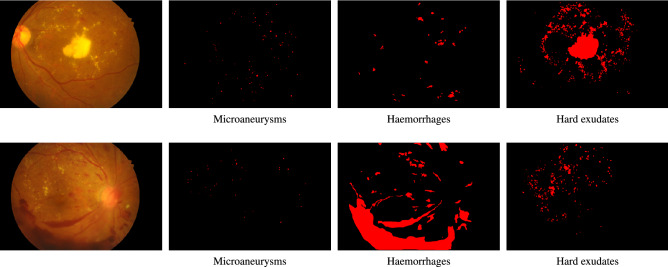
Sample images and their corresponding pixel-level lesion annotations from the IDRiD dataset.

The Indian Diabetic Retinopathy Dataset (IDRiD)^31^ contains fundus images captured during real clinical examinations in an eye clinic in India using a Kowa VX fundus camera. The captured images have a 50° field of view with a resolution of $4288\times 2848$. The images are separated into 3 parts, corresponding to 3 different learning tasks and accompanied by the respective types of ground-truth. The first part is designed for the development of segmentation algorithms and contains 81 images (54 train set–27 test set) with pixel-level annotations of DR lesions (microaneurysms, haemorrhages, hard and soft exudates) and the optical disk. The second part corresponds to a DR grading task and contains 516 images divided into train set (413 images) and test set (103 images) with DR and Diabetic Macular Edema (DME) severity grades. Finally, the third part corresponds to a localization task and contains 516 images with the pixel coordinates of the optic disk center and fovea center (again split in a 413 train and 103 test set). Sample images from the segmentation part along with their corresponding lesion annotations are given in Fig. 5.

## Results

### Implementation details

We perform extensive experiments to assess the proposed model’s ability to detect rDR in fundus images. For training, we use the gradable (according to^15^) images in the Kaggle-EyePACS training split, while for evaluation we use the Kaggle test split and Messidor-2 datasets. Before any training, we randomly sample 3000 images from the Kaggle training and set them aside for use as a validation set. For training, we use the standard binary cross-entropy loss. Assuming that $\hat{p}_{\mathrm{data}}$ denotes the empirical data-label distributions defined by the training set, the cross-entropy loss is defined as:

3 $$\begin{aligned} {\mathscr {L}} = \mathop {-{\mathbb {E}}}_{X,y_{\mathrm{rdr}} \sim \hat{p}_{\mathrm{data}}} \big [y_{\mathrm{rdr}} \log (p_{\mathrm{model}}(y_{\mathrm{rdr}} | X)\big ] \end{aligned}$$

**Figure 6:**
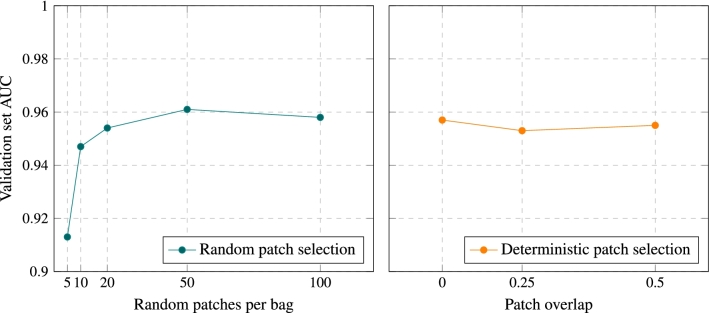
AUC scores on the Kaggle validation set for the random patch selection policy (left) and the deterministic patch selection policy (right). In the case of random patch selection, we examine the effect of *K* (number of randomly selected patches per image) on validation performance, while for the deterministic patch selection we examine the effect of *t* (patch overlap ratio).

Raghu et al.^36^ showed that transferring weights from the natural to the medical image domain is not necessary to achieve high classification performance. However, it does speed up convergence and thus, we initialized the function $\phi$ (ResNet-18) with pre-trained ImageNet weights. During training we perform image augmentation by randomly shifting, flipping, scaling and rotating the image and by randomly applying small perturbations to its brightness, contrast, hue and saturation. We convert the image to its bag of patches representation by randomly selecting $K=50$ random patches from the pool of all possible image patches. This value was selected based on its superior classification performance (Fig. 6) on the validation set. At test time, instead, we extract patches on a regular grid with a patch overlap rate of 0.75. The function $\phi$ transforms an input patch to a feature vector of length $M=128$, while an attention module of dimension $L=32$ is used to produce the pooled image representation *z*. We use the Adam optimizer with a base learning rate of $3\cdot 10^{-4}$ for 60 epochs and the suggested $b_1, b_2$ parameters. After training, we keep the model instance that achieved the highest performance on the validation set.

### Classification experiments

For measuring classification performance we use the *Area Under the Receiver Operating Curve (ROC AUC)*, a metric commonly used in the related literature. We also compute the model’s sensitivity and specificity metrics at the operating points of high specificity ($>0.9$) and high sensitivity ($>0.9$). We report the performance metrics of a 5 model ensemble on both the Kaggle-EyePACS test set and Messidor-2 in Table 2, along with a comparison to related works. Ideally, such a comparison would include methods that were trained and evaluated on the same datasets and under the same conditions. However, there is a widely inconsistent use of datasets and evaluation metrics throughout the rDR classification literature^9^, with different methods using different datasets for training/testing or even custom training/test splits. For instance, Voets et al.^15^ and Zhou et al.^26^ use Kaggle splits that favor significantly larger training sets, as opposed to the official splits in which the test set is $\sim$ 1.5 times larger than the training set. After taking this into consideration, we compare our method to the most prominent works of the rDR literature that use the same publicly-available datasets. More specifically, in the first part of Table 2 we include methods that were trained and evaluated on Kaggle-EyePACS using the officially provided training-test splits, while the second part is reserved for methods that used custom splits. In the third part we include methods that were trained with any dataset and evaluated on Messidor-2 per image (i.e. one prediction per image). This part is also segmented into 3 sub-parts that group methods depending on which Messidor-2 grades they used. Finally, the fourth part contains methods that were trained on any dataset and evaluated on Messidor-2 on a per-subject basis (i.e. a single prediction after taking into account 2 images per subject).

**Table 2:** Comparison of classification performance between the proposed approach and the most notable works in the related literature for the Kaggle-EyePACS and Messidor-2 datasets.

Training	Evaluation	Method	AUC	High sensitivity		High specificity	
				Sens	Spec	Sens	Spec
Kaggle-train (all)	Kaggle-test (all)	MIL attention	**0.961**	0.951	0.763	0.851	0.950
		Leibig et al.^17^	0.927	NA		NA	
		Rakhlin et al.^37^	0.920	0.920	0.720	0.800	0.920
		Pires et al.^16^	0.946	NA		NA	
		Graham et al.^14^	0.951	NA		NA	
		Quellec et al.^28^	0.954	NA		NA	
Kaggle (gradable)	Kaggle (gradable)	Voets et al.^15^	0.941	0.899	0.838	0.834	0.901
Kaggle-train (custom split)	Kaggle-train (custom split)	Zhou et al.^26^	0.925	NA		NA	
Kaggle-train (gradable)	Kaggle-test (gradable)	MIL attention	**0.957**	0.951	0.753	0.843	0.951
Kaggle-train (gradable)	Messidor-2 (3 graders)	MIL attention	0.976	0.954	0.845	0.856	0.953
Kaggle-train (all)		MIL attention	0.974	0.952	0.838	0.867	0.950
Kaggle-train (custom split)	Messidor-2 (UIowa per image)	Voets et al.^15^	0.800	0.737	0.697	0.697	0.764
Kaggle-train (all)		Rakhlin et al.^37^	0.970	0.990	0.710	0.870	0.920
EyePACS		Gargeya et al.^19^	0.940	NA		NA	
Enriched EyePACS	Messidor-2 (7 graders)	Gulshan et al.^13^	**0.990**	0.961	0.939	0.870	0.985
Kaggle-train (gradable)	Messidor-2 (UIowa per subject)	MIL attention	0.972	0.958	0.861	0.816	0.950
Kaggle-train (all)		MIL attention	0.972	0.968	0.839	0.837	0.950
Private		Abramoff et al.^8^	0.935	0.968	0.594	NA	
Private		Abramoff et al.^38^	**0.980**	0.968	0.870	NA	

For Kaggle we report separately methods that use the official training/test split (first part of the table) from works that use custom splits or subsets of the full set (second part of the table). Sensitivity/specificity values for the operating points of high sensitivity/specificity are reported when available. Similarly, we separate results on Messidor-2 depending on the DR grades they were obtained with. The highest AUC in each part of the table is given in bold.

As we can see, in terms of rDR classification performance, our method perform on par with the state-of-the-art literature. In fact, when evaluated on the Kaggle test set, our approach outperforms the alternatives in terms of AUC score, even those that use larger training sets, achieving an AUC of 0.961 on the official Kaggle test set. On the Messidor-2 per-image task, our method is only outperformed by the work of Gulshan et al.^13^, who used a much larger and better annotated training set (> 100k images annotated by a panel of specialists, in contrast to our $\sim$ 28k images annotated by a single expert). However, due to the variance in the Messidor-2 grades that were used by the considered methods in this experiment, many performance comparisons may be biased and should be interpreted with caution. It should be noted here that we did not evaluate our approach on the Messidor-2 per image task using the UIowa grades, because only the per-subject rDR grades were made publicly available by this source (and not the per-image ones). Finally, when evaluating on the Messidor-2 per subject task, our method achieves competitive performance (AUC of 0.972) but clearly falls behind Abramoff et al.^38^ who achieve an AUC of 0.980.

**Figure 7:**
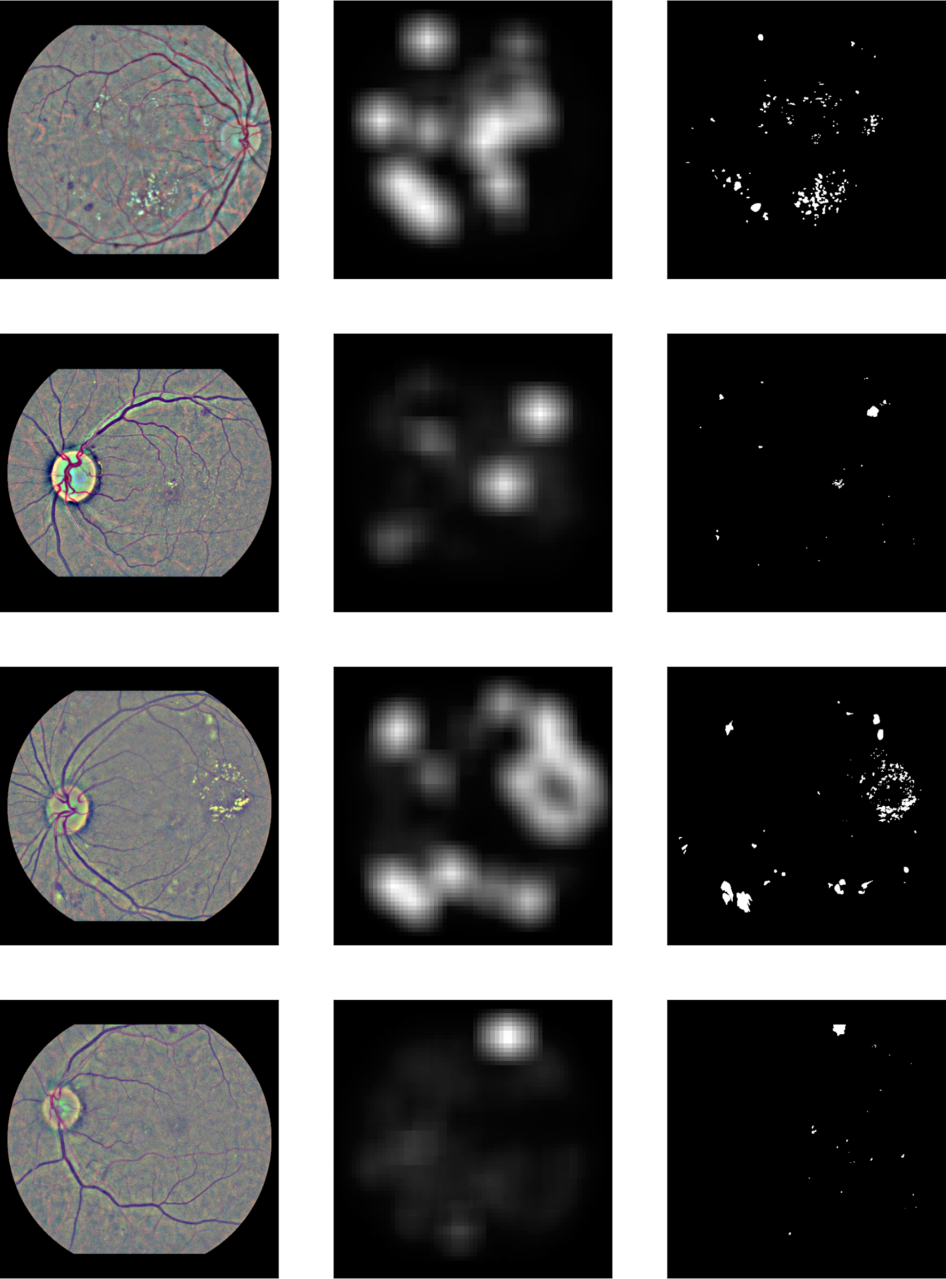
Examples of the attention heatmaps (middle column) produced by the model for 4 IDRiD images (left column), presented side-by-side with the available pixel-level lesion ground-truth (right column). For the purposes of easier presentation, the ground-truth images (right column) are a combination of all the available lesion annotations (microaneurysms, haemorrhages and exudates).

### Attention heatmap evaluation

By taking advantage of the per-patch attention coefficients, we can construct heatmaps that pinpoint the fundus areas that the model focused on for arriving at a prediction. Examples of such heatmaps for both correct and incorrect predictions in the Messidor-2 dataset are presented in Fig. 4, while examples for IDRiD images alongside their corresponding lesion ground-truth are presented in Fig. 7. As we can see by inspecting these 2 figures, the model seems to attend more to patches that contain artifacts resembling DR lesions, such as microaneurysms, haemorrhages and soft or hard exudates. Rather than relying on visual inspection of the produced heatmap, we would like to evaluate its validity more quantitatively. To that end, we employ the first part of the IDRiD dataset that contains 80 images with detailed, pixel-level lesion annotations. Based on these images, we can verify the correlation between per-patch attention weight and lesion existence that Figs. 4 and 7 suggests. To do so, we conduct two experiments. In the first experiment, we use the attention weight assigned to each patch as a predictor of whether the patch contains any DR lesion. We compute the AUC and the Area Under Precision Recall Curve (AUPRC) of the per-patch attention weight against a binary label that denotes lesion existence. For the purposes of this experiment, we extract patches on a regular grid with high patch overlap ($87.5\%$), in order to enrich the pool of patches. Any patch that contains even a tiny amount of lesion (at least 1 pixel according to the available ground-truth) is assigned to the positive class and otherwise to the negative class. We report the aforementioned classification metrics against different labels, corresponding to microaneurysms, haemorrhages, exudates (we have merged soft and hard exudates in a single class, since only a fraction of IDRiD images contain soft exudate ground-truth), as well as all lesions combined, in Table 3. As we can see, the attention weight achieves good performance, especially when the target label is produced by considering all lesions together. This is to be expected, as the training procedure allows the model to focus on what it considers relevant to the task at hand and, as a result, it does not explicitly learn to prioritize a specific lesion type over some other. However, if we go one step further and extrapolate the lesion predictions from the patch level to the pixel level (Table 4), we can see that the patch-level attention weights cannot serve as a reliable pixel-level lesion predictor. Again, this is to be expected considering that we have assigned the same prediction to all pixels in a specific patch (since, in reality, we cannot produce true per-pixel predictions). Yet, if we keep in mind that the model has not been trained with any fine-grained lesion information and that any lesion localization ability has emerged automatically from training using the attention mechanism, this is a fair result.

**Table 3:** Resulting AUC and AUPRC scores when using the attention weight of a patch as a binary predictor of lesion presence.

Lesion	Negative patches	Positive patches	AUC	AUPRC
Microaneurysms	163.735	96.185	0.760	0.611
Haemorrhages	180.655	79.265	0.745	0.528
Exudates	162.637	97.283	0.749	0.591
Any lesion	103.362	156.558	0.800	0.869

We report scores against different lesion types individually, as well as all lesions combined. Results are produced using images of the IDRiD dataset for which pixel-level lesion annotations are available.

**Table 4:** Resulting AUC and AUPRC scores when using the attention weight of a patch as a binary predictor of lesion presence for each pixel.

Lesion	Negative pixels	Positive pixels	AUC	AUPRC
Microaneurysms	20.948.589	22.931	0.803	0.003
Haemorrhages	20.760.978	210.542	0.726	0.021
Exudates	20.733.012	238.508	0.748	0.023
Any lesion	20.499.643	471.877	0.744	0.047

We report scores against different lesion types individually, as well as all lesions combined. Results are produced using images of the IDRiD dataset for which pixel-level lesion annotations are available.

**Figure 8:**
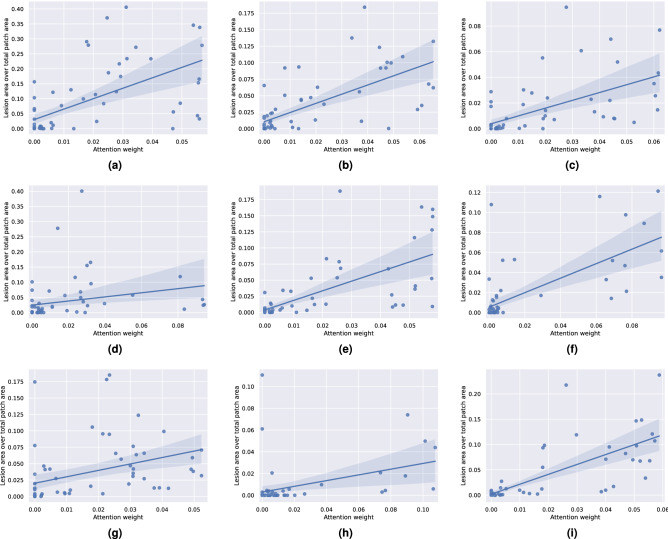
Example scatterplots of the attention weight assigned by the model to each patch versus the percentage of the patch area that contains lesions. The plots are computed using images from the IDRiD dataset, for which detailed per-pixel lesion annotations for microaneurysms, haemorrhages, soft and hard exudates are available. A linear regression fit is estimated for each image and overlayed on the plot to highlight the linear trend.

In the second experiment, we are interested in verifying whether the attention weight of a patch depends on the amount of lesions it contains. To that end, we compute the scatterplots of the attention weights versus the percentage of the area of a patch that corresponds to lesions (i.e. how many pixels in a given patch have been annotated as belonging to any lesion category). We present such plots for 9 images of the IDRiD dataset in Fig. 8. Based on these figures, we find that there seems to be a positive linear correlation between the magnitude of the attention weight and the lesion dominance in the patches of an image. Nevertheless, the existence of outliers suggests that in some cases the size of the lesion may not be the most dominant factor in the attention distribution, and other factors, like smaller lesions but with more characteristic shapes, could be deemed more interesting by the model.

## Discussion

The development of methods for automated DR grading is one of the most popular applications of machine learning in recent years. According to a recent survey paper^9^, there is an exponential rise of interest in the field after 2015, with over 50 papers in 2018 and 70 papers in 2019 using deep learning. Yet, despite the vast growth, there are no standardized benchmarks for DR algorithms and, as a result, there is no easy way to compare fairly with other works. This is made worse by the inherent inter- (and intra-) grader variability human experts themselves exhibit^6^ when manually grading DR. As different works often produce their own DR grades for a dataset in cooperation with their eye specialists, one must keep in mind that even when comparing with more standardized datasets such as Messidor-2, there might be discrepancies between the DR grades used. In this work we made a conscious effort to facilitate easy comparisons with future work by employing standard training/test splits and the official DR grades associated with each dataset.

With respect to classification performance, we have demonstrated that the attention-based MIL approach is a viable alternative to the usual pipelines for rDR detection. More specifically, it proved resilient against the noisy setting of the Kaggle-EyePACS dataset, where, to the best of our knowledge, it achieved the highest AUC on the single image prediction task. In the cross-dataset testing scenario, it achieved an AUC of 0.976 on Messidor-2, which is comparable to the relevant state-of-the-art (AUC of 0.99) and often superior to that of other models trained on larger datasets.

**Figure 9:**
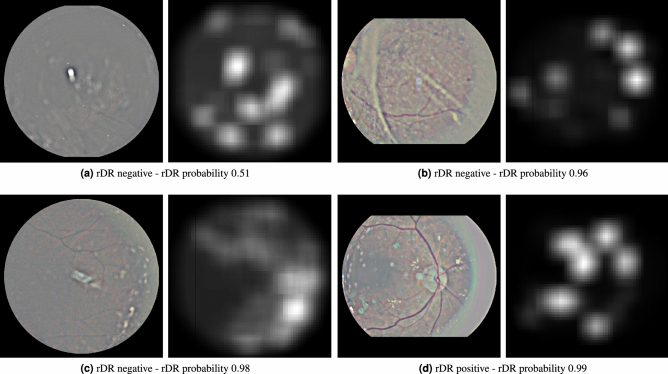
Visual examples of attention heatmaps for non-gradable (according to the estimates by Voets et al.^15^) images of Kaggle-test. The proposed model achieved an AUC of 0.948 on the non-gradable subset of the Kaggle-test set.

A rather interesting side-result, stems from the fact that we can achieve better performance when using the full Kaggle set (0.961 AUC), compared to when using only the gradable part (0.957 AUC). This could lead to doubts about the quality of the gradability estimates provided by Voets et al.^15^ and call into question the claim that non-experts can accurately judge the gradability of a retinal image. In fact, when evaluated on the non-gradable subset of the Kaggle-test set, our model reached an AUC of 0.948. We also provide some visual examples non-gradable Kaggle-test images and their corresponding attention heatmap in Fig. 9.

Instead of extracting image patches deterministically (i.e. using a pre-defined grid), our model was trained on pools of randomly sampled patches. Naturally, this modification greatly improved the training time, as an image can be represented by as little as 50 patches, while extracting patches over a grid with $50\%$ overlap yields 225 $64\times 64$ patches for a $512\times 512$ image. Most interestingly, it also slightly increased the model’s performance on the validation set (Fig. 6). This can be explained by thinking the random patch policy as a form of implicit data augmentation: each time a specific image is used for training, the model will, with high probability, see a new bag of patches encoding, owing to the image being represented by a relatively small random subset of all possible patches.

Apart from detection accuracy, model intrepretability is a common requirement in medical applications of machine learning, as it allows human experts to make sense of why the model makes some prediction and not the opposite. Thus, interpretability is key in the acceptance of any proposed machine learning solution in the field of eye care. Methods for incorporating interpretability to DR prediction models have already been suggested. For instance, Sundararajan et al.^39^ proposed a generic method for attributing the prediction of a network to its inputs. The method was tested on DR prediction models and has shown great value in assisting ophthalmologists grade new images^40^. In a similar vain, our method outputs a heatmap that contains the attention weights assigned to each image region during inference. This attention heatmap emerges as a natural by-product of the model and consequently does not require additional gradient computations, as is the case in alternative interpretability approaches for deep networks such as Grad-cam^41^ or Axiomatic Attribution^39^. It can be used alongside the rDR probability prediction, to inform medical experts of the particular fundus artifacts that led to that prediction. Furthermore, it can help machine learning practitioners understand their model’s failures and find ways to overcome them. For instance, we can see that in false positive predictions (images without rDR that are predicted as rDR) our model usually focuses on regions that contain either tiny spots that resemble microaneurysms (Fig. 4f), yellow lesions that resemble hard exudates (Fig. 4e) or bright cotton-wool-like artifacts (Fig. 4d). Bringing these findings to the attention of eye specialists could facilitate a better understanding as to why the model mistakes these artifacts for lesions, as well as a potential strategy to counter such factors of confusion.

One limitation of the proposed approach is the need for large patch overlap during inference. While this is not an issue during training (due to the random patch sampling), it can pose a memory constraint on the system’s deployment. For classification an overlap value of 0.75 (which results in 841 patches per image) achieves good performance and can be handled relatively easily. Nevertheless, for constructing the auxiliary attention heatmaps larger values are necessary in order to have more fine-grained and pleasing visualizations. As a measure of scale, the images in Fig. 4 were produced using a patch overlap of 0.875, the largest value that a GPU system with 8GB of memory (NVidia 1070Ti) could handle.

Finally, an interesting line of investigation for future work, would be to question the utility of using a CNN designed for large resolution images (such as ResNet-18) to process patches of quite smaller dimensions ($64\times 64$). In fact, while for this work we opted for a clearly over-parameterized approach, it will be interesting to test whether a model designed to work with smaller resolutions (e.g. a ResNet-20 that is designed for $32\times 32$ images), performs equally well or even better. Another topic of future research will be to examine how to utilize pixel-level DR lesion annotations, typically available in very small amounts due to the tediousness of producing them, in order to improve the predictive performance and lesion heatmap quality. This remains an open problem, as DR grading and DR lesion localization methods have remained more or less orthogonal, with very few published works on the topic^42^.

## Conclusions

We introduced a system that detects referable diabetic retinopathy in fundus images, by extracting local information from each image patch separately and combining it with an attention mechanism. In our experiments, the proposed system achieved high classification performance, making it competitive with state-of-the-art works that were trained in larger and better annotated data. Aside from its high predictive value, our system can inherently produce a heatmap of the regions on which its decision was based, thus aiding in the interpretation of its predictions.
